# Inferior Vena Cava Collapsibility Index Can Predict Hypotension and Guide Fluid Management After Spinal Anesthesia

**DOI:** 10.3389/fsurg.2022.831539

**Published:** 2022-02-17

**Authors:** Ting-ting Ni, Zhen-feng Zhou, Bo He, Qing-he Zhou

**Affiliations:** ^1^Department of Anesthesiology, Ningbo No.7 Hospital, Ningbo, China; ^2^Department of Anesthesiology, Hangzhou Women's Hospital, The Affiliated Women's Hospital of Hangzhou Normal University, Hangzhou, China; ^3^Department of Gynecology, Ningbo No.7 Hospital, Ningbo, China; ^4^Department of Anesthesiology, The First Affiliated Hospital, Jiaxing University, Jiaxing, China

**Keywords:** inferior vena cava collapsibility index, spinal anesthesia, hypotension, inferior vena cava ultrasound, intravascular volume

## Abstract

**Purpose:**

We hypothesized that inferior vena cava collapsibility index (IVCCI)-guided fluid management would reduce the incidence of postspinal anesthesia hypotension in patients undergoing non-cardiovascular, non-obstetric surgery.

**Methods:**

A receiver operating characteristic (ROC) curve was used to determine the diagnostic value of IVCCI for predicting hypotension after induction of spinal anesthesia and calculate the cut-off value. Based on the cut-off variation value, the following prospective randomized controlled trial aimed to compare the incidence of postspinal anesthesia hypotension between the IVCCI-guided fluid administration group and the standard fluid administration group. Secondary outcomes included the rate of vasoactive drug administration, the amount of fluid administered, and the incidence of nausea and vomiting.

**Results:**

ROC curve analysis revealed that IVCCI had a sensitivity of 83.9%, a specificity of 76.3%, and a positive predictive value of 84% for predicting postspinal anesthesia hypotension at a cut-off point of >42%. The area under the curve (AUC) was 0.834 (95% confidence interval: 0.740–0.904). According to the cut-off variation value of 42%, the IVCCI-guided group exhibited a lower incidence of hypotension than the standard group [9 (15.3%) vs. 20 (31.7%), *P* = 0.032]. Total fluid administered was lower in the IVCCI-guided group than in the standard group [330 (0–560) mL vs. 345 (285–670) mL, *P* = 0.030].

**Conclusions:**

Prespinal ultrasound scanning of the IVCCI provides a reliable predictor of hypotension following spinal anesthesia at a cut-off point of >42%. IVCCI-guided fluid management before spinal anesthesia can reduce the incidence of hypotension following spinal anesthesia.

## Introduction

Spinal anesthesia is a safe and reliable method frequently used in various lower abdominal, orthopedic, and obstetric operations. It has advantages such as the rapid onset of action, cost-effectiveness, ease of administration, and relatively fewer side effects. Hypotension and bradycardia are the most common side effects of spinal anesthesia (1, 2) and may lead to several adverse outcomes including coronary ischaemia and delirium (3, 4). Most of the available prediction models used to estimate the risk factors for hypotension are based on non-modifiable factors such as age >40 years, emergency surgery, history of hypertension, and baseline systolic blood pressure <120 mmHg (1, 5, 6). It is necessary to identify readily available variables to help anesthesiologists recognize patients with modifiable risk levels, such as those with haemodynamic impairment. However, assessment of the intravascular volume status is challenging for clinicians. Different techniques such as pulmonary arterial catheter, PiCCO, and Vigileo have been described to assess preload among other elements of haemodynamic status. However, their universal use remains an object of ongoing debate due to financial restrictions, relatively high complication rates, and invasiveness in most operations (7).

Many methods such as preventive empirical volume loading or prophylactic vasopressors, have been used to lower the incidence of hypotension following spinal anesthesia (3, 8). However, intravenous volume preload carries the potential for volume overload, particularly in patients with cardiac disease (9). Furthermore, due to different definitions of hypotension and diverse patient populations, the effect of volume preload on prevention of hypotension is still controversial. Many studies have identified sonographic determination of inferior vena cava (IVC) collapsibility index (IVCCI) as non-invasive, and easy technique for evaluating volume status. Recent guidelines from the American Society of Echocardiography support the general use of IVCCI in assessing volume status (10). The Previous study has also shown that IVCCI can be determined by bedside ultrasound and correlated with hypovolemia during spontaneous breathing (11). Preau et al. (12) indicate that IVCCI is a simple predictor of volume overload in sepsis-related acute circulatory failure patients. Moreover, operators can practice this method with little experience in echocardiography (13, 14). To date, the predictive value of IVC ultrasound examination remains inconclusive (15, 16).

The present prospective study aimed to evaluate the power of preoperative IVCCI for predicting the incidence of hypotension following spinal anesthesia. According to this research, a cut-off value was accepted as a positive fluid response. We hypothesized that using IVCCI-guided fluid management instead of standard care for patient-adapted fluid treatment before spinal anesthesia would significantly reduce the incidence of hypotension following spinal anesthesia in patients undergoing non-cardiovascular, non-obstetric surgery.

## Methods

The present study was approved by the Ethical Committee of Ningbo No.7 Hospital. The study was pre-registered at http://www.chictr.org.cn/index.aspx (ChiCTR1900027848). Written informed consent was obtained from participants before enrollment.

### Patients

Adult patients aged 18 to 65 years and having American Society of Anesthesiologists (ASA) physical status grades I–II who were scheduled for non-cardiovascular, non-obstetric surgery under spinal anesthesia at Ningbo No.7 Hospital. Exclusion criteria were pre-existing hypotension (defined as systolic arterial pressure <90 mmHg or mean arterial pressure <60 mm Hg), severe cardiovascular disease [unstable angina or ejection fraction <40%, implanted pacemaker/cardioverter, decompensated heart failure, and elevated pulmonary arterial pressure >40 mmHg (13, 17)], contraindication for spinal anesthesia, canal stenosis, pregnant patients, body mass index (BMI) >30 kg/m^2^, or failure to perform spinal anesthesia.

### IVC Ultrasonography

All IVC measurements were performed in the supine position before administering spinal anesthesia using a Sonosite Edge (Fujifilm Sonosite Inc., Bothell, WA, USA) ultrasonography machine. All IVC measurements were performed by a single anesthesiologist fully trained in ultrasound who had at least 3 years of experience in this field.

The IVC was carried out using a paramedian long-axis view via subcostal approach as stated by the method described by the American Society of Echocardiography (10). Doppler waveform and phasic collapse with respiration were used to differentiate the IVC. Measurements of the IVC diameter were obtained in M-mode imaging performed 2 to 3 cm distal to the right atrium in the long-axis subcostal view (18). Three scans were performed for each patient and the entire IVC scan procedure required <10 min. The maximum (dIVCmax) and the minimum (dIVCmin) anteroposterior diameters of the IVC at the end of expiration and inspiration were taken during the same respiratory cycle. The IVCCI was calculated using the following formula: IVCCI = (dIVCmax –dIVCmin)/dIVCmax × 100% (19).

### Anesthesia Management

All patients fasted for 8 h before the surgery. Standard monitoring (electrocardiogram, blood pressure measurements, and peripheral oximeter readings) was performed once in the operating room. No fluid load was administered to any of the patients before spinal anesthesia. Heart rate (HR) and mean blood pressure (MBP) were measured three times before anesthesia with an interval of 2 min between measurements, and the average values were recorded.

An anesthesiologist who was not involved in the study administered spinal anesthesia with a 25-gauge Quincke needle. After the L3-L4 interspace was confirmed by radiographic imaging, a dose of 12 to 15 mg of 0.5% plain bupivacaine (depending on the surgery and the patient's constitution) was injected intrathecally for 10 s when the free flow of cerebrospinal fluid was obtained. After injection, patients were immediately positioned in the supine position for 30 min before the surgery. Meanwhile, non-invasive blood pressure measurements were performed every minute, and other vital parameters were recorded continuously during the period following spinal anesthesia. The sensory block level was evaluated with a pinprick test by an anesthetist who was not involved in the study, with the aim of a T8-T6 level block.

An episode of hypotension was defined as a decrease in the MBP by more than 30% of the baseline value or any recorded period of MBP <60 mmHg during the period following spinal anesthesia. Severe hypotension was defined as MBP <55 mmHg. Episodes of hypotension were treated using 5 mL/kg of crystalloids infused within 15 min. After 2 min of persistent hypotension or MBP <55 mmHg, appropriate vasoactive drug (ephedrine 5 mg, phenylephrine 100 μg, atropine 0.5 mg) was administered every 2 min depending on improvement in the patient's condition. Any complications such as nausea and vomiting, discomfort, shivering, or allergic reactions were noted and managed accordingly.

## Prediction of Hypotension

The current observational study was conducted in Ningbo No.7 Hospital from January 2020 to March 2020 after obtaining written informed consent from all included patients. The present study used the ROC curve to evaluate the power of preoperative IVCCI for predicting hypotension after induction of spinal anesthesia.

### Statistical Analysis

A pilot study including 32 patients, which utilized the receiver operating characteristic (ROC) curve, detected an area under the curve (AUC) of 0.7 for IVCCI and 33% of the patients experienced spinal anesthesia-induced hypotension. The sample size was calculated using the difference of 0.2 between the AUC of 0.5 computed using the null hypothesis and the AUC of 0.7 calculated using the prediction of hypotension following spinal anesthesia. Based on this result, a sample of 95 patients achieved a power of 90% to detect a statistically significant difference (at a level of 0.05).

The ROC curve was used to determine the diagnostic value of IVCCI for predicting hypotension after induction of spinal anesthesia. Multivariable logistic regression analysis was performed to detect the association between IVCCI and hypotension following spinal anesthesia. Variables including demographic characteristics, ASA physical status, baseline MBP, dIVCmax, and IVCCI potentially associated with hypotension following spinal anesthesia or those that had a *p*-value < 0.10 in the univariate analyses were included in the multivariate logistic regression analyses (1, 6, 20–22). Multicollinearity diagnostic tests were carried out by variance inflation factor (VIF). By convention, multicollinearity is considered present if the VIF of one variable is at least 10 (23).

## Results

### Patient Data and Hemodynamic Data

**Figure 1:**
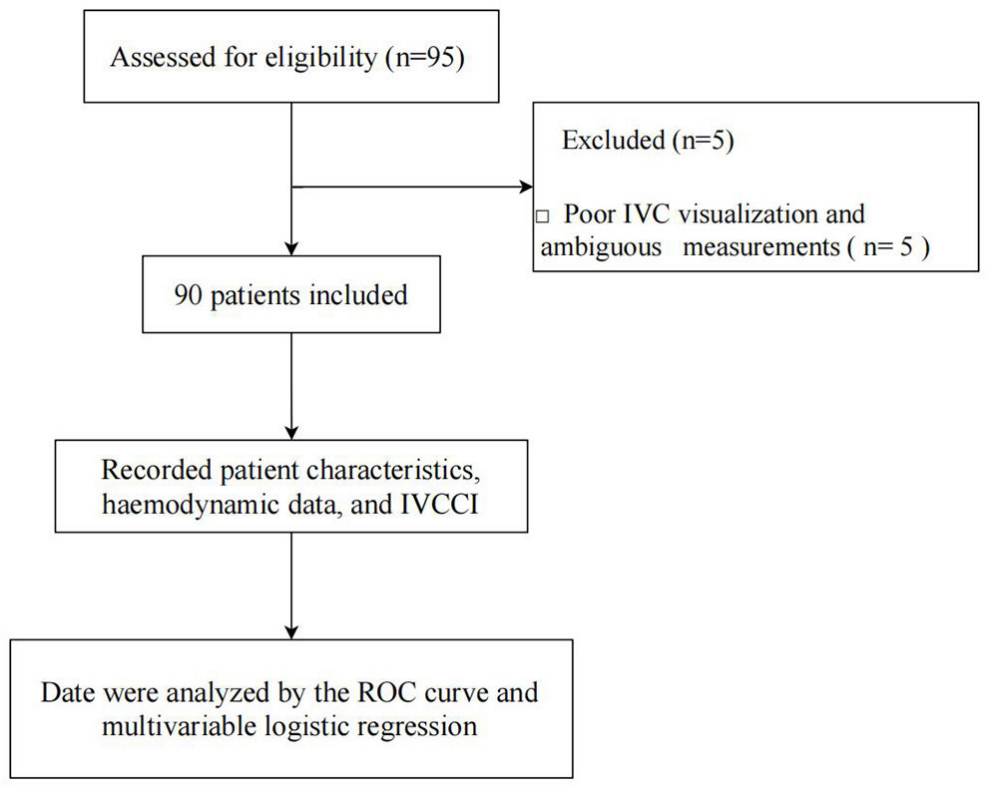
Flow diagram outlining the study procedure.

Ninety-five patients were enrolled in the present study from January 2020 to March 2020. Five patients were excluded due to poor IVC visualization and ambiguous measurements. Thus, 90 patients were included in the final analysis (Figure 1). After spinal anesthesia, 31 (34.4%) patients developed hypotension. Among these, seven patients received ephedrine and two received phenylephrine for severe or hypotension lasting more than 2 min. Three patients were administered atropine for sinus bradycardia. A significant difference was observed in baseline MBP between patients who developed hypotension and those with hemodynamic stability (*P* = 0.001). Patients who developed hypotension had a smaller dIVCmax (*P* = 0.0001) and a higher IVCCI (*P* < 0.0001). Demographic and perioperative characteristics are presented in Table 1.

**Table 1:** Demographic and perioperative characteristics of patients.

**Variable**	**Combined (*n* = 90)**	**Hypotensive group (*n* = 31)**	**Normotensive group (*n* = 59)**	***P*** **-Value**
Age, years	52.0 ± 11.0	54.4 ± 9.9	50.7 ± 11.0	0.107
BMI, kg/m^2^	23.2 ± 2.6	23.6 ± 2.1	23.1 ± 2.8	0.515
Female sex, *n* (%)	42 (46.7%)	15 (48.3%)	27 (45.8%)	0.813
Bupivacaine, mg	13.1 ± 1.6	13.0± 1.5	13.2 ± 1.6	0.494
ASA (I/II)	56/34	19/12	37/22	0.878
Baseline MBP, mmHg	96.8 ± 6.2	99.7 ± 5.1	95.3 ± 6.2	0.001
Baseline HR, beats/min	77.8 ± 14.2	79.4 ± 12.6	76.9 ± 14.9	0.434
History of hypertension, *n* (%)	20 (22.2%)	6 (19.4%)	14 (23.7%)	0.635
Use of b-blockers, *n* (%)	7 (7.8%)	2 (6.5%)	5 (8.5%)	0.733
Use of ACE-inhibitors, *n* (%)	11 (12.2%)	3 (9.7%)	8 (13.6%)	0.593
Surgery duration, min	87.5 ± 18.4	85.4 ± 18.9	88.5 ± 18.2	0.449
dIVCmax, mm	12.9 ± 2.5	11.9 ± 2.0	13.4 ± 2.5	0.009
IVCCI, %	40.7 ± 6.9	46.1 ± 5.1	37.0 ± 6.0	<0.0001
**Type of surgery**				
Lower limb surgery, *n* (%)	26 (28.9%)	8 (25.8%)	18 (30.5%)	0.640
Lower abdominal surgery, *n* (%)	24 (26.7%)	8 (25.8%)	16 (27.1%)	0.894
Gynecological surgery, *n* (%)	11 (12.2%)	4 (12.9%)	7 (11.9%)	0.886
Urology, *n* (%)	29 (32.2%)	11 (35.5%)	18 (30.5%)	0.631

*Data are expressed as means ± SDs, medians [interquartile ranges], or numbers (%). BMI, body mass index; ASA, American Society of Anesthesiologists; dIVCmax, the maximum diameter of IVC; dIVCmin, the minimum diameter of IVC; HR, heart rate; MBP, mean blood pressure; IVCCI, collapsibility index of inferior vena cava*.

### ROC Curve Analysis for All Patients

**Figure 2:**
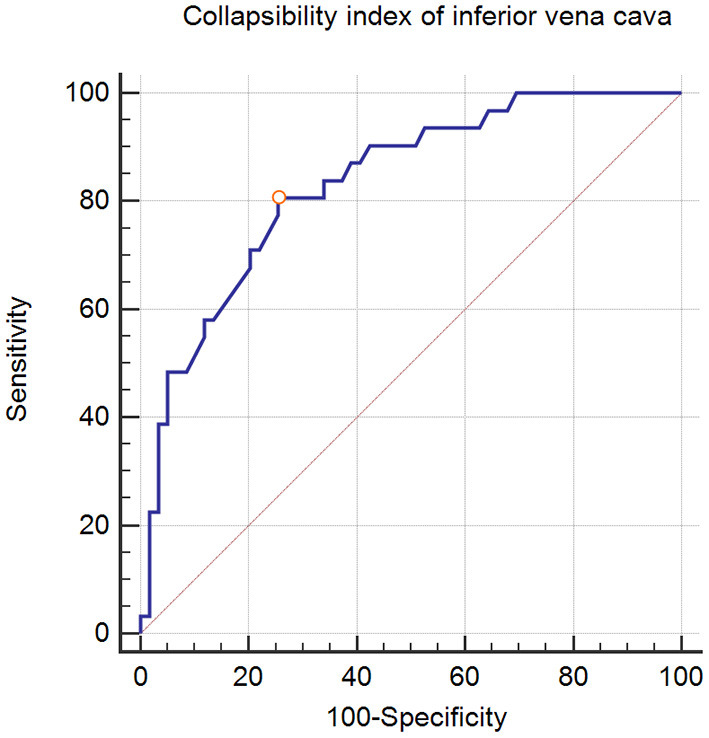
Receiver operating characteristic curves showing the ability of collapsibility index of inferior vena cava to predict hypotension following spinal anesthesia. The circle on the curves indicate the optimal cut-off values determined by maximizing the Youden index.

ROC curve analysis showed that IVCCI had a sensitivity of 83.9%, a specificity of 76.3%, and a positive predictive value of 84% for predicting hypotension following spinal anesthesia at a cut-off value of >42%. The AUC was 0.834 (95% confidence interval: 0.740–0.904) (Figure 2). Hypotension occurred in 6 of the 49 patients with IVCCI < 42%.

### Regression Analysis

After adjusting for age, BMI, ASA physical status, baseline MBP, and dIVCmax, IVCCI was a strong predictor of spinal anesthesia-induced hypotension (*P* < 0.0001). The variables in the model showed the VIF around 0.860 to 1.163. Patients with higher IVCCI were more likely to develop hypotension after spinal anesthesia, with an odds ratio of 1.283 (1.137–1.448) (Table 2).

**Table 2:** Multivariable logistic regression analysis of 90 patients for postspinal anesthesia hypotension.

**Predictors**	**Regression coefficient**	**Odds ratio**	**95% CI**	***P*** **-Value**
Age	0.036	1.037	0.982–1.094	0.192
BMI	0.039	1.040	0.807–1.341	0.762
ASA(I/II)	−0.212	0.809	0.233–2.805	0.738
Baseline MBP	0.087	1.091	0.975–1.220	0.128
dIVC max	−0.231	0.793	0.615–1.023	0.084
IVCCI	0.249	1.283	1.137–1.448	<0.0001

*BMI, body mass index; ASA, American Society of Anesthesiologists; dIVCmax, the maximum diameter of IVC; IVCCI, collapsibility index of inferior vena cava; 95% CI, 95% confidence interval of partial correlation coefficients*.

## IVCCI-Guided vs. Standard Fluid Administration

The following prospective randomized controlled trial aimed to compare the incidence of postspinal anesthesia hypotension between the IVCCI-guided fluid administration group and the standard fluid administration group. The study was designed according to the CONSORT 2010 (Supplementary File 1). After getting informed consent, we randomly allocated eligible participants to either the standard or IVCCI-guided groups from April 2020 to October 2020. Microsoft Excel's random number generator was used for computer-generated randomization and allocations were concealed using sequentially numbered opaque sealed envelopes.

IVC ultrasound was conducted before spinal anesthesia in patients from both groups. Patients allocated to the standard group received a therapy based on Ningbo No.7 Hospital historic fluid administration data (5 mL/kg of crystalloids over 15 min). Patients in the IVCCI-guided group received fluid therapy based on our preliminary study. Patients with positive results (IVCCI more than the cut-off value) received a bolus of 5 mL/kg of crystalloids over 15 min. Subsequently, the IVCCI was reassessed again. Identical fluid boluses were administered (5 mL/kg of crystalloids) until a non-fluid responder pattern was observed. In the IVCCI-guided group, patients with an IVCCI less than the cut-off value didn't receive any infusion.

The primary outcome measure of the subsequent prospective randomized controlled trial was the incidence of hypotension following spinal anesthesia between the groups. Secondary outcomes included the quality of vasoactive drug administration, the amount of fluid administered (total, pre-anesthesia, and post-anesthesia), and the incidence of nausea and vomiting.

### Statistical Analysis

For the primary outcome, a clinically significant difference in the incidence of hypotension following spinal anesthesia between the study groups was set at 12% according to our pilot trial (incidence of hypotension following spinal anesthesia was 18% in the IVCCI-guided group and 30% in the standard group). Based on this calculation, we concluded that 120 patients would be needed to achieve a probability (power) of 80% with an α level of 0.05 (analyses were conducted with MedCalc 13.0; MedCalc Software Ltd., Ostend, Belgium).

Continuous data were expressed as mean ± standard deviation and variables were compared between the study groups using independent Student's *t*-test. A two-tailed Mann–Whitney U test was used to evaluate the significance of the non-parametric data. We analyzed the associations among discrete variables using the chi-squared test or Fisher's exact test. All statistical analyses were performed using IBM SPSS Statistics for Windows (version 22.0; IBM Corp., Armonk, NY, USA) and MedCalc for Windows (version 13.0; MedCalc Software Ltd., Ostend, Belgium). Two-tailed *p*-values < 0.05 were considered statistically significant.

## Results

### Patients

**Figure 3:**
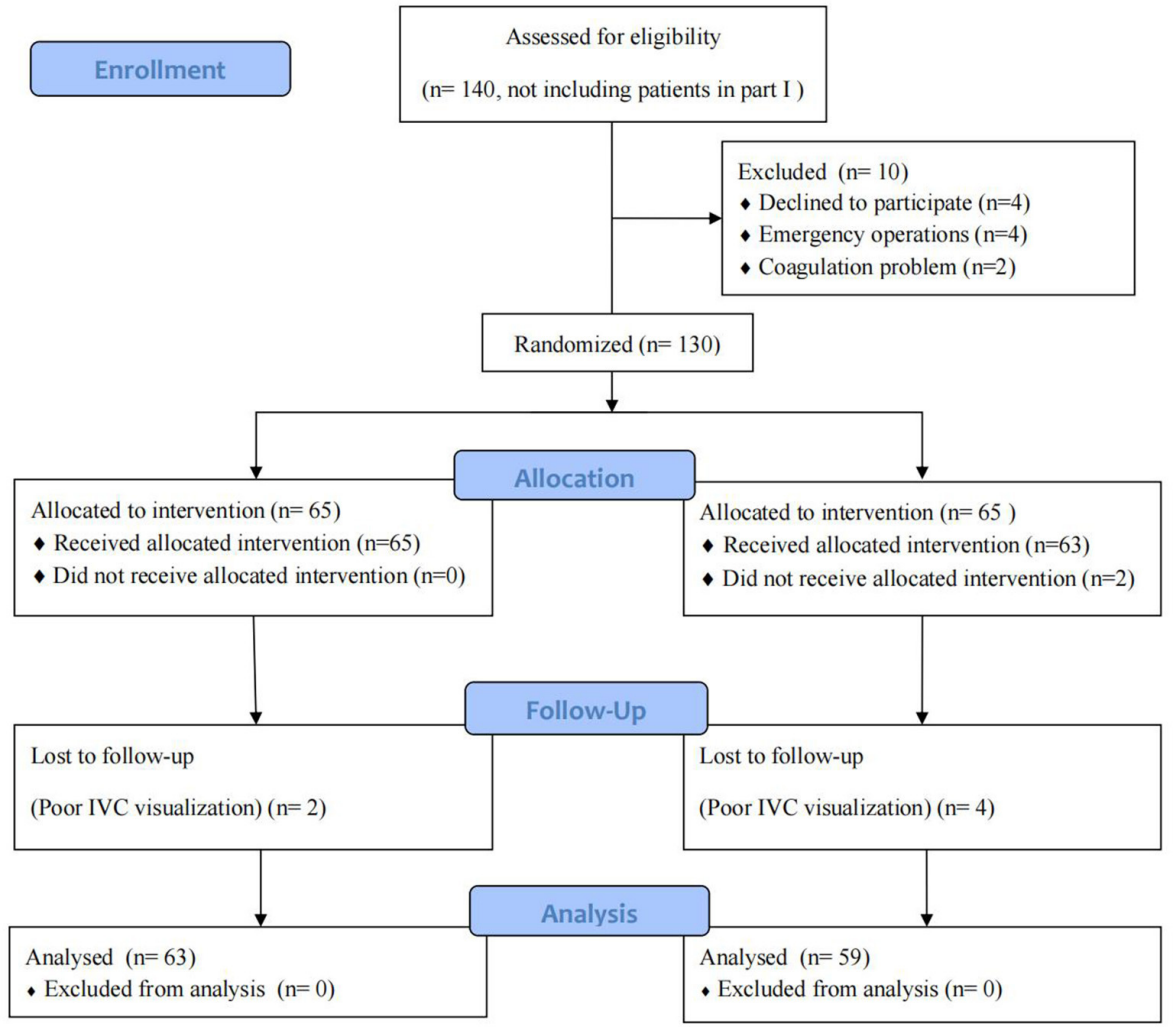
Flow diagram outlining the enrollment and randomization study procedures.

Between April 2020 to October 2020, 140 patients were enrolled. Among these, 10 patients met the exclusion criteria, six were excluded after randomization due to poor IVC visualization, and two withdrew consent. Fifty-nine patients were randomized into the IVCCI-guided group and 63 patients were randomized into the standard group (Figure 3). The population and surgical characteristics were similar in both groups (Table 3).

**Table 3:** Patient's baseline data in IVCCI-guided group and the standard group.

**Variable**	**Standard group (*n* = 63)**	**IVCCI-guided group (*n* = 59)**	***P*** **-Value**
Age, y	54.2 ± 15.0	51.7 ± 12.4	0.194
BMI, kg/m^2^	22.3 ± 2.1	21.8 ± 2.4	0.273
ASA (I/II)	24/39	29/30	0.218
Weight, kg	62.8 ± 8.1	65.0 ± 9.3	0.162
Female sex, *n* (%)	28 (44.4%)	27 (45.8%)	0.884
Bupivacaine, mg	13.0 ± 1.3	13.1 ± 1.5	0.456
Block level, segment	15.8 ± 0.8	15.7 ± 0.8	0.517
Baseline MBP, mmHg	92.1 ± 7.6	89.9 ± 7.1	0.101
Baseline HR, beats/min	76.0 ± 10.7	78.4 ± 9.8	0.200
History of hypertension, *n* (%)	16 (25.4%)	18 (30.5%)	0.529
Use of b-blockers, *n* (%)	5 (7.9%)	7 (11.9%)	0.467
Use of ACE-inhibitors, *n* (%)	10 (15.9%)	11 (18.6%)	0.685
Surgery duration, min	79.6 ± 11.8	82.3 ± 13.2	0.679
dIVCmax, mm	11.9 ± 2.0	12.3 ± 1.9	0.261
IVCCI, %	45.2 ± 4.3	47.7 ± 3.9	0.331
IVCCI > 42%, *n* (%)	35 (55.6%)	37 (62.7%)	0.422
**Type of surgery**			
Lower limb trauma surgery, *n* (%)	20 (31.7%)	17 (28.8%)	0.725
Lower abdominal surgery, *n* (%)	15 (23.8%)	16 (27.1%)	0.675
Gynecological surgery, *n* (%)	11 (17.5%)	8 (13.6%)	0.553
Urology, *n* (%)	17 (27.0%)	18 (30.5%)	0.667

*Data are expressed as means ± SDs, medians [interquartile ranges], or numbers (%). BMI, body mass index; ASA, American Society of Anesthesiologists; dIVCmax, the maximum diameter of IVC; dIVCmin, the minimum diameter of IVC; HR, heart rate; MBP, mean blood pressure; IVCCI, collapsibility index of inferior vena cava*.

### Primary Outcome

The overall incidence of hypotension following spinal anesthesia was 23.8%. The IVCCI-guided group showed a lower incidence of hypotension than the standard group [9 (15.3%) vs. 20 (31.7%), *P* = 0.032]. Hypotension occurred in 5 of the 50 patients with IVCCI < 42 % in two groups, including 2 in the standard group and 3 in the IVCCI-guided group. Severe hypotension after spinal anesthesia occurred in 14.8% (18 out of 122) of the patients, with no statistically significant difference between the groups [10.2% (6 out of 59) in the IVCCI-guided group vs. 19.0% (12 out of 63) in the standard group, *P* = 0.167] (Table 4).

**Table 4:** Patient's outcomes in IVCCI-guided group and the standard group.

**Variable**	**Standard group (*n* = 63)**	**IVCCI-guided group (*n* = 59)**	***P*** **-Value**
Hypotension, *n* (%)	20 (31.7%)	9 (15.3%)	0.032
Severe hypotension, *n* (%)	12 (19.0%)	6 (10.2%)	0.167
Use of vasopressors, *n* (%)	17 (27.0%)	7 (11.9%)	0.036
Nausea, *n* (%)	13 (20.6%)	4 (6.8%)	0.027
Vomiting, *n* (%)	7 (11.1%)	2 (3.4%)	0.103
Preanesthesia fluid amount, ml	330 (280–365)	323 (0–530)	0.766
Postanesthesia fluid amount, ml	0 (0–335)	0 (0–0)	0.015
Total fluid amount, ml	345 (285–670)	330 (0–560)	0.030

### Secondary Outcomes

**Figure 4:**
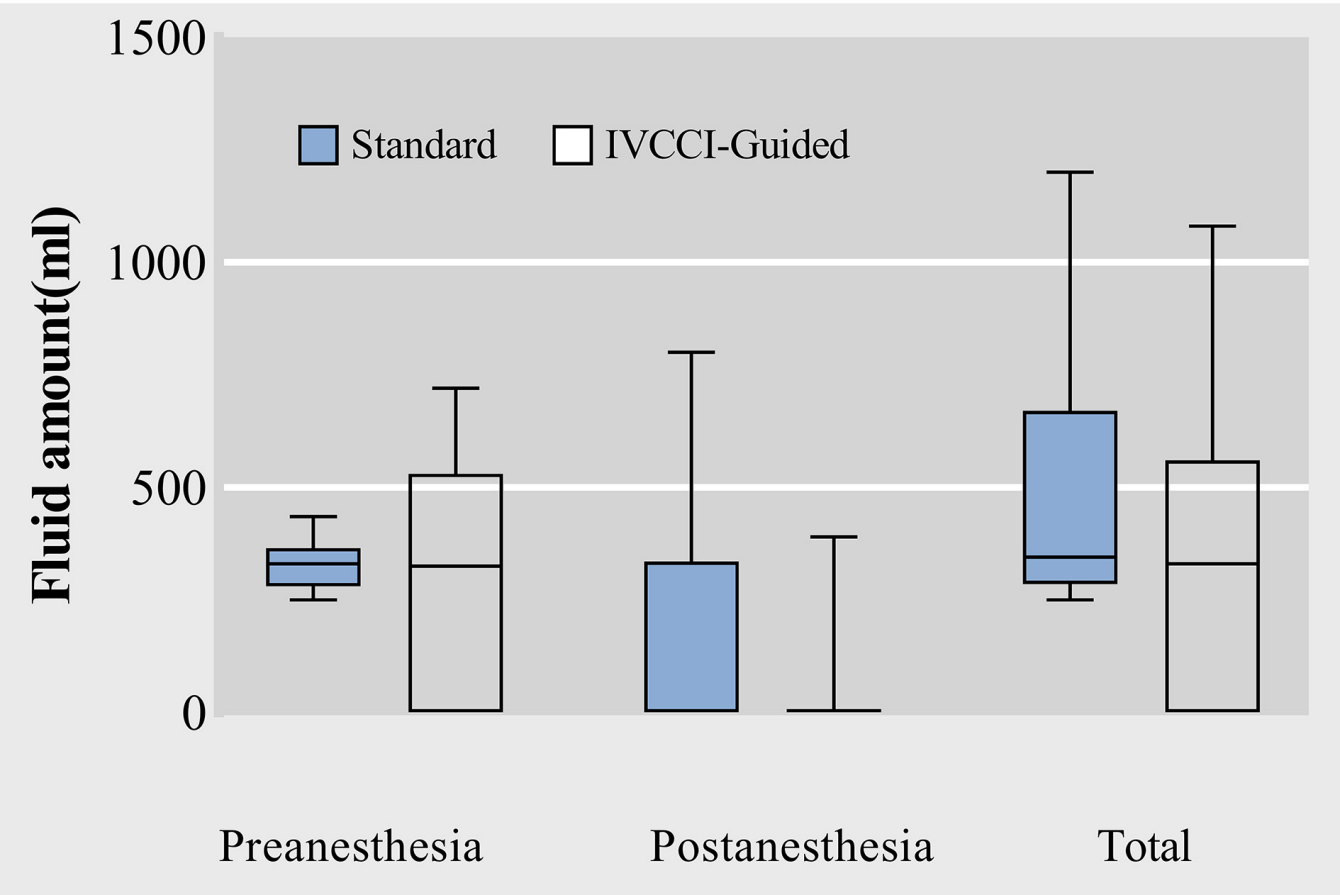
Box plots of raw data for preanesthesia, postanesthesia and total fluid amount administrated. Data are median (central line) and interquartile range (box margins).

The global rate of vasoactive drug administration was 20.5%. Altogether, 11.9% of the patients (*n* = 7) in the IVCCI-guided group and 27.0% of the patients (*n* = 17) in the standard group required a vasoactive drug at least once and the difference between the groups was significant (*P* = 0.036). Postoperative nausea occurred in 6.8% (4 out of 59) of the patients in the IVCCI-guided group and in 20.6% (13 out of 63) of the patients in the standard group (*P* = 0.027). Total fluid administered in the IVCCI-guided group was lower than that in the standard group [330 (0–560) mL vs. 345 (285–670) mL, *P* = 0.030]. In the IVCCI-guided group, 37 patients with IVCCI > 42 % had fluid responsiveness before spinal anesthesia (Figure 4). Nineteen patients (32.2%) required one bolus of 5 mL/kg crystalloid, while 18 patients (30.5%) received two boluses adjustment of fluid administration. The IVCCI-guided group showed a lower fluid administration than the standard group after spinal anesthesia [0 (0–0) mL vs. 0 (0–335) mL, *P* = 0.015] (Table 4).

## Discussion

The present study found that IVCCI determination using ultrasound before spinal anesthesia could predict subsequent hypotension with a cut-off value of 42%. Moreover, IVCCI-guided fluid administration reduced the incidence of hypotension following spinal anesthesia by 16% compared with standard fluid therapy.

In a recent retrospective analysis, Monk et al. (24) described an association between perioperative hypotension and 30-day postoperative mortality in patients undergoing non-cardiac surgery. Despite this evidence, most anesthesiologists still use blood pressure and HR as primary measurements for haemodynamic monitoring during surgery even in high-risk patients (25). IVC ultrasound is a non-invasive, quickly learned, and conducted approach that is often used to predict fluid responsiveness for guiding fluid treatment in intensive care settings and emergencies. Consequently, the inclusion of bedside IVC ultrasound will help identify patients requiring fluid optimization.

Accumulating evidence has revealed that IVC diameter is a valuable indicator of volume status (26) and respiratory variation helps assess fluid responsiveness (27). A previous study found a greater IVCCI, especially with a small initial reference diameter of the IVC, suggested a low-volume status (28). Zhang and Critchley (13) indicated that preoperative ultrasonographic IVCCI measurements could predict the occurrence of hypotension after induction of general anesthesia at a cut-off value of 43%. Salama and Elkashlan (15) also found a similar optimal cut-off value of 44.7% for predicting hypotension after induction of spinal anesthesia. Our results showed that IVCCI was an independent predictor of hypotension following spinal anesthesia after adjusting for age, BMI, ASA physical status, baseline MBP, and dIVCmax, which is consistent with previous studies (8, 15, 29).

We observed a reduction in the incidence of spinal anesthesia-induced hypotension in the IVCCI-guided group. Unlike the standard group in which all patients received one bolus of 5 mL/kg crystalloid, 62.7% of the patients in the IVCCI-guided group needed adjustment of fluid administration and 30.5% of the patients received two boluses of fluid administration, which suggested that IVCCI measurement could identify an individual patient's need for boluses of fluid preload. Another study by Ceruti et al. (30) compared IVCCI-guided fluid administration and no fluid preload infusion before spinal anesthesia. They found a lower incidence of hypotension in the IVCCI-guided fluid administration group. We hypothesized that the development of hypotension after spinal anesthesia might be related to increased IVC collapsibility. Preoperative IVCCI measurement was a reliable predictor of hypotension after induction of spinal anesthesia, wherein an IVCCI >42% was the threshold. However, Hypotension was observed in 6 of the 49 patients with IVCCI <42%. It is known that spinal anesthesia can inhibit the sympathetic nerves, leading to peripheral vascular dilation and a low-volume status. So IVCCI may be increased after spinal anesthesia, even if the measurement was <42% before anesthesia. Our study suggested that the lower incidence of hypotension was associated with decreased fluid administration after spinal anesthesia. The total fluid administered was lower in the IVCCI-guided group than in the standard group. The present study also showed a reduction in the administration of vasoactive drugs and the incidence of nausea in the IVCCI-guided group, which may be related to the lower incidence of hypotension after spinal surgery.

Sonographic measurement of the IVC diameters and calculation of the IVCCI provide a reliable non-invasive tool for guidance of intraoperative fluid and vasopressor management in high-risk patients (31). Due to the simplicity of IVC ultrasound for perioperative use and its non-invasive nature, IVCCI-based fluid therapy strategy for patients undergoing surgery under spinal anesthesia should be encouraged. IVCCI measurements should be performed before spinal anesthesia to screen patients with risk of spinal-induced hypotension, particularly elderly patients and those suspected of hypovolemia. Further, specifically targeted research are desirable better to investigate this particular group, including ASA IV-V patients, optimizing the volume and avoiding water overload.

The present study has some limitations. We did not measure IVC collapsibility after spinal anesthesia. Thus, changes in the IVCCI after spinal anesthesia were not included in this research. More studies are needed to define the impact of postspinal anesthesia hemodynamic status on the changes in IVCCI. Salama and Elkashlan (15) found that the IVC/aorta diameter index is more helpful than IVCCI in predicting hypotension following spinal anesthesia. However, this index was not included in our study. Moreover, the lack of blinding of patients to the allocation might have led to biases. However, biases could be reduced in part by randomization and blinding of the statistician to data evaluation. In addition, we studied only the patients with ASA grade I–II and those aged 18 to 65 years to analyse our hypothesis in a natural clinical setting. Another limitation is that the regression analysis needs the assumption of linearity for covariates but may not hold in reality. The complex relationship between predictors and response variables is usually unknown in many instances. Furthermore, machine-learning algorithms that do not require strict assumptions regarding data structure should be synthesized to improve predictive accuracy further. In addition, these results originated from research where clinical assessments were performed only under one fluid management strategy. We cannot exclude that other strategies may influence the predictive value of IVCCI for predicting the incidence of hypotension after spinal anesthesia. Finally, all measurements were done by a single anesthesiologist to increase the accuracy of the size. There may be intra-observer variability in these measurements. Differences in the mean measurements would be more accurate if this possible variability was evaluated.

## Conclusion

IVCCI determined using ultrasound before spinal anesthesia is a reliable predictor of the incidence of hypotension following spinal anesthesia at a cut-off point of >42%. IVCCI-guided fluid management before spinal anesthesia can reduce the incidence of spinal anesthesia-induced hypotension.

## Data Availability Statement

The raw data supporting the conclusions of this article will be made available by the authors, without undue reservation.

## Ethics Statement

The studies involving human participants were reviewed and approved by Ethical Committee of Ningbo No.7 Hospital. The patients/participants provided their written informed consent to participate in this study.

## Author Contributions

Q-hZ: writing-review and editing. T-tN: methodology, formal analysis, writing-original draft, and writing-review and editing. Z-fZ: data curation and writing-review and editing. BH: data curation and project administration. All authors read and approved the final manuscript.

## Funding

This study was supported by the Science and Technology Project of Ningbo, Zhejiang Province (2020Y36). The funders had no role in study design, data collection and analysis, decision to publish, or manuscript preparation.

## Conflict of Interest

The authors declare that the research was conducted in the absence of any commercial or financial relationships that could be construed as a potential conflict of interest.

## Publisher's Note

All claims expressed in this article are solely those of the authors and do not necessarily represent those of their affiliated organizations, or those of the publisher, the editors and the reviewers. Any product that may be evaluated in this article, or claim that may be made by its manufacturer, is not guaranteed or endorsed by the publisher.

## Supplementary Material

Supplementary File 1 CONSORT 2010 Checklist.
